# The effect of herbivory on pollinators: a revisited meta-analysis

**DOI:** 10.1093/aob/mcaf258

**Published:** 2025-10-16

**Authors:** Stephanie Haas-Desmarais, Bastien Castagneyrol, Luis Abdala-Roberts, Christopher J Lortie, Anna Traveset, Xoaquín Moreira

**Affiliations:** Department of Biology, York University, 4700 Keele St., Toronto, Ontario, Canada M3J 1P3; BIOGECO, INRA, University of Bordeaux, Cestas 33610, France; Universidad Nacional Autónoma de México, Escuela Nacional de Estudios Superiores-Unidad Mérida, Carretera Mérida-Tetiz Km. 4.5, Ucú, Yucatán 97357, México; Department of Biology, York University, 4700 Keele St., Toronto, Ontario, Canada M3J 1P3; Mediterranean Institute for Advanced Studies (CSIC), Global Change Research Group, Cl Miquel Marques 21, Esporles, Balearic Islands 07190, Spain; Mision Biologica de Galicia (MBG-CSIC), Apartado de Correos 28, Pontevedra, Galicia 36080, Spain

**Keywords:** Herbivory, pollinators, plant–animal interactions, floral traits, reproductive success, indirect interactions

## Abstract

**Background and Aims:**

Plant–herbivore and plant–pollinator interactions are closely interconnected through their combined influence on plant reproduction, involving both direct and indirect (plant-mediated) effects between these consumer groups. Although these dynamics have been investigated for nearly three decades and were previously synthesized in a meta-analysis, rapid growth in the field in recent years warrants an updated quantitative assessment.

**Methods:**

We extend the most recent synthesis by incorporating primary studies published between 2018 and 2023, nearly doubling the dataset from 88 to 171 studies and increasing the number of independent observations from 568 to 1348. We reanalysed the effects of both natural and simulated herbivory on floral traits, pollinator visitation and plant reproductive output, expanding previous damage categories to include stem damage and mixed-tissue damage – defined here as damage affecting multiple plant tissues simultaneously (e.g. grazing that impacts both leaves and flowers).

**Key Results:**

Plant damage significantly reduced floral traits, pollinator attraction and reproductive success. These effects varied with both the type of damage and the tissue affected, with their interaction strongly moderating plant responses. Natural damage to leaves and flowers in most cases reduced floral traits, pollinator visitation and reproduction (except floral traits in the case of flower damage). By contrast, root and mixed damage had no significant effects. Simulated damage, on the other hand, also influenced responses: damage to flowers and stems only reduced floral traits, while damage to leaves reduced pollinator attraction. Importantly, our updated analysis corroborates some trends but also overturns earlier findings: whereas previous work suggested no impact of simulated herbivory, we now detect significant negative effects, and natural floral damage, once considered neutral or in some cases positive for reproduction, is revealed to be detrimental.

**Conclusions:**

These findings demonstrate that herbivory alters plant–pollinator interactions in trait- and tissue-specific ways, providing new insights into the ecological and evolutionary consequences of plant–herbivore–pollinator linkages.

## INTRODUCTION

A large body of research has demonstrated that herbivores can significantly reduce plant fitness through direct effects on growth, survival and reproduction (e.g. Marquis, 1984; Maron, 1998; Karban and Agrawal, 2002; Mesa *et al*., 2019). However, it has become increasingly clear that herbivores also influence plant fitness indirectly by altering interactions with other organisms, particularly pollinators. One compelling example involves herbivory-induced changes to floral traits that, in turn, affect pollinator behaviour and ultimately plant reproductive success (Bronstein *et al*., 2007; Kessler and Halitschke, 2009; Jones and Agrawal, 2017). Herbivores can directly damage flowers, reducing floral size and symmetry and thereby decreasing pollinator attraction (Mothershead and Marquis, 2000; Botto-Mahan *et al*., 2011; Tsuji and Ohgushi, 2018). Beyond floral tissues, damage to vegetative organs such as leaves, stems and roots can also deplete the resources available for floral development and nectar or pollen production (Bruinsma *et al*., 2014; Burkle and Runyon, 2016; Haas and Lortie, 2020), with downstream consequences for pollinator visitation (Mitchell *et al*., 2004; Parachnowitsch *et al*., 2019; Brunet *et al*., 2021). Yet, herbivory is not always detrimental; in some cases, plants exhibit reproductive overcompensation – producing more flowers in response to damage – potentially increasing pollinator visitation (Strauss *et al*., 2001; Irwin *et al*., 2004; Schiestl *et al*., 2014).

Such interplay between herbivores and pollinators has attracted increasing attention over the past two decades, with several syntheses summarizing the ecological consequences of these interactions (Lucas-Barbosa, 2016; Jacobsen and Raguso, 2018; Moreira *et al*., 2019; Haas and Lortie, 2020; Underwood *et al*., 2020). However, the field is rapidly expanding, with the number of published studies on plant–herbivore–pollinator interactions rising sharply in recent years. This growth is probably driven by increasing recognition of the complexity of multitrophic interactions, advances in molecular and analytical techniques allowing a detailed study of chemical and ecological interactions, and heightened interest in understanding how these interactions respond to global change (Underwood *et al*., 2020; McPeek *et al*., 2022). Consequently, earlier meta-analyses – such as that by Moreira *et al*. (2019) – already require comprehensive updates to incorporate new evidence and refine our understanding. Replication and synthesis are foundational principles of scientific progress (Baker, 2016; Fraser *et al*., 2020). Meta-analyses, in particular, are designed to be reproducible and updatable (Gurevitch *et al*., 2018), and extensions of previous syntheses are warranted as new studies accumulate, new analytical methods emerge or past conclusions require reassessment (Jennions *et al*., 2013). Revisiting and updating previous syntheses can therefore increase confidence in prior findings or reveal new patterns previously obscured by limited data.

Given the growing urgency to understand species interactions under global change (Schleuning *et al*., 2020; Fricke *et al*., 2022), we present an updated meta-analysis that more than doubles the dataset compiled by Moreira *et al*. (2019). Our analysis examines the effects of both natural and simulated herbivory on floral traits, pollinator attraction and plant reproductive success, with a broader scope that now includes damage to stems and mixed tissues (e.g. from grazing). We specifically test whether increased sample size changes the magnitude or direction of previously observed effects and explore the importance of tissue type and herbivory mode in mediating plant–pollinator dynamics. This updated synthesis addresses a central question in plant–animal interaction theory and contributes to a more nuanced understanding of the ecological processes shaping plant fitness via shared interactions with herbivores and pollinators.

## MATERIAL AND METHODS

### Data collection

We compiled studies from the meta-analyses conducted by Moreira *et al*. (2019) (*n* = 88) and the literature review by Haas and Lortie (2020) (*n* = 59). After removing duplicate studies between the two sources (*n* = 12), we screened the remaining studies from Haas and Lortie (2020) to ensure they met the inclusion criteria established by Moreira *et al*. (2019). Specifically, studies were required to (1) report data on pollinator responses and/or floral traits of animal-pollinated plant species in relation to herbivore damage, and (2) provide either raw data (via tables, supplementary material, or third-party repositories such as Dryad) or treatment-level means accompanied by a measure of variability (standard deviation, standard error, or variance) and sample sizes. Based on these criteria, 15 studies were excluded. The final dataset from these two sources included 120 studies comprising 888 independent study cases, of which 566 originated from Moreira *et al*. (2019).

In July 2023, we updated the literature search using the Web of Science, applying the search terms used in both Moreira *et al*. (2019) and Haas and Lortie (2020). From Moreira *et al*. (2019), we used the search string: ‘(Plant OR tree OR shrub) AND (herbivore OR herbivores OR herbivorous) AND (flower OR floral OR nectar OR inflorescence OR pollinator OR pollination)’, excluding records published before June 2018 (the date of their original search). From Haas and Lortie (2020), we used: ‘(herbivor AND pollinat) OR floriv* OR (foliv* AND pollinat*) OR (herbivor* AND flower*) OR (foliv* AND flower)’, excluding records published before October 2019. These two search strings were consolidated and executed simultaneously in Web of Science to minimize duplication. The search yielded 894 publications. After applying the same inclusion criteria described earlier, we identified 51 new studies comprising 460 additional study cases. These were added to the existing dataset, resulting in a final total of 171 studies and 1348 independent study cases.

In addition to extracting data on pollinator attraction and floral traits, we also compiled information on plant fitness, as it is often correlated with pollinator visitation (Underwood *et al*., 2020). For all studies included in Moreira *et al*. (2019) and Haas and Lortie (2020), as well as newly added studies, we recorded the following variables: plant species identity and life history (i.e. annual or perennial), type of herbivory (natural or simulated) and the plant tissue attacked (flowers – sepals and petals – leaves, roots). We also collected data on floral traits – including flower number, floral size, nectar volume or concentration, and flowering phenology – and on pollinator attraction, measured as pollinator abundance or diversity, number of visits or flowers visited, and duration of visits. Plant reproductive success was quantified using metrics such as seed or fruit number and weight, and seed viability. Furthermore, we incorporated two additional categories of plant tissue damage not considered in the original analyses: stem damage and mixed damage. We are unable to separate mixed damage into finer categories (flowers/stems, flowers/leaves, stems/leaves or all above-ground tissues) because the source studies generally did not report this level of detail. Additionally, vertebrate grazers can simultaneously feed on multiple tissues, making it difficult to attribute damage to a specific type or combination of tissues.

### Statistical analyses

Our statistical analyses followed the framework established by Moreira *et al*. (2019). For each study case, we calculated effect sizes using Hedges’ *g* (Hedges, 1981), which accounts for small sample bias, implemented via the *metafor* package (Viechtbauer, 2010) in R v.4.4.2 (R Core Team, 2024). Negative effect sizes indicate that herbivory had a detrimental effect on the response variable, whereas positive values reflect a beneficial effect. We began by conducting an omnibus test incorporating all study cases, to assess the overall impact of herbivory (regardless of damage type or response variable) on plant traits and functions. Specifically, we tested whether the overall mean effect size across all cases differed significantly from zero. We then assessed whether total heterogeneity in effect sizes could be explained by the following moderators: response variable (floral traits, pollinator attraction, plant reproduction), plant tissue damaged (flowers, leaves, roots, stems or mixed), and type of damage (natural or simulated). While analysing floral traits (e.g. flower number/size versus nectar traits) and pollinator attraction metrics (abundance vs. visitation duration) separately could reveal interesting patterns, the available sample sizes for these data subsets were too small to allow for reliable analyses.

Each moderator was first tested independently to determine its main effect. Subsequently, all moderators were included in a fully crossed design to examine their interactive effects on plant- and pollinator-related responses to herbivory. All analyses were conducted using multilevel mixed-effects meta-analysis models, incorporating random effects of replicate analysis nested within study ID to account for non-independence and within-study variability. Additionally, to address dependence among study cases that shared control treatments within a study, we applied a variance–covariance matrix of the sampling errors, which models the correlation between non-independent effect sizes (Gleser and Olkin, 2009). Finally, we conducted sensitivity and publication bias analyses to assess the robustness of our results (see Supplementary Data Appendix S1 for details).

## RESULTS

The sample size of study cases increased across nearly all moderator combinations (i.e. each unique combination of damage type, tissue affected, and response variable), with the sole exception of simulated root damage on floral traits. Increases ranged from 20 % (for the effect of natural root damage on reproductive success) to 920 % (for the effect of simulated leaf damage on reproduction; Table 1). Across all response variables, the overall mean effect of herbivory was negative and statistically significant (mean effect size ± s.e. = −0.20 ± 0.04; *z* = −5.42, *P* < 0.0001). We also detected substantial heterogeneity among study cases (τ² = 0.26, *Q*_T_ = 5275.72, *P* < 0.0001), with most of the variation attributable to differences between rather than within studies (*I²* = 71.4 %).

**Table 1.** Effects of damage type (natural or simulated herbivory) on each tissue (roots, leaves, flowers, stems and mixed) and the number of study cases (*k*) in the original dataset (Moreira *et al*., 201*9*) versus the updated dataset (including studies published since 2018). Negative (−) and non-significant (n.s.) effects are indicated.

	Original dataset		Updated dataset	
	Effect	*k*	Effect	*k*
**Floral traits**				
Natural herbivory				
Roots	n.s.	9	n.s.	12
Leaves	(−)	129	(−)	169
Flowers	(−)	33	n.s.	59
Stem		0	n.s.	56
Mixed		0	n.s.	149
Simulated herbivory				
Roots	n.s.	2	n.s.	2
Leaves	n.s.	49	n.s.	77
Flowers	n.s.	38	(−)	46
Stem		0	(−)	20
Mixed		0	n.s.	11
**Pollinator attraction**				
Natural herbivory				
Roots	n.s.	6	n.s.	12
Leaves	(−)	126	(−)	185
Flowers	(−)	23	(−)	81
Stem		0	n.s.	12
Mixed		0	n.s.	24
Simulated herbivory				
Leaves	n.s.	3	(−)	9
Flowers	n.s.	30	n.s.	44
Mixed		0	n.s.	12
**Plant reproduction**				
Natural herbivory				
Roots	n.s.	10	n.s.	12
Leaves	(−)	69	(−)	112
Flowers	n.s.	17	(−)	57
Stem		0	n.s.	14
Mixed		0	n.s.	44
Simulated herbivory				
Leaves	n.s.	5	n.s.	51
Flowers	n.s.	17	n.s.	46
Stem		0	n.s.	29
Mixed		0	n.s.	3

**Fig. 1.**
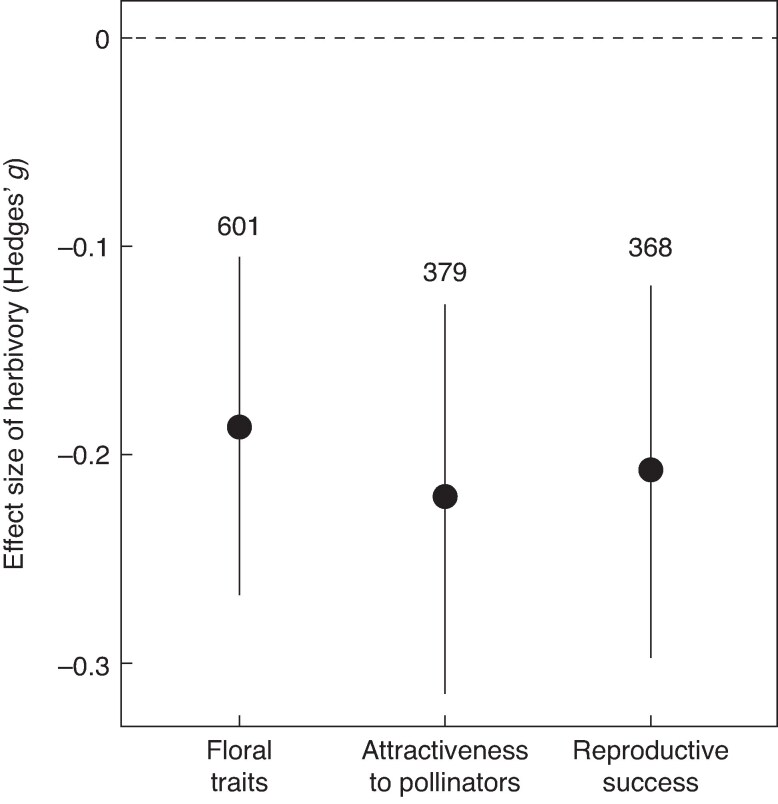
Mean effect sizes (Hedges’ *g*) of herbivory on floral traits, pollinator attraction, and plant reproductive success. Dots and error bars represent model estimates and their 95 % bias-corrected confidence intervals (CIs). The number of study cases (*k*) is indicated above the error bars. The vertical dashed line at zero represents the null hypothesis (no difference between control and herbivore-damaged plants). Effects are considered significant when the 95 % CI does not overlap zero.

**Fig. 2.**
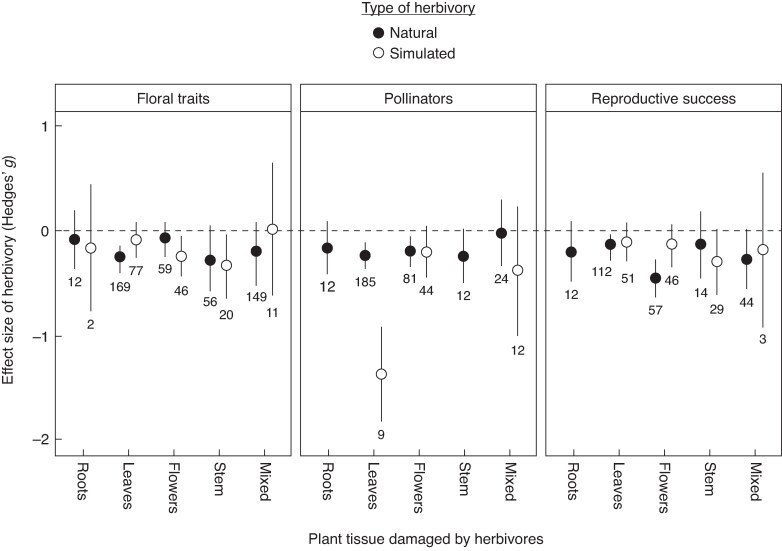
Mean effect sizes (Hedges’ *g*) of herbivory, considering the tissue damaged (roots, leaves, flowers, stems or mixed) and type of damage (natural vs. simulated), on floral traits, pollinator attraction and plant reproductive success. Dots and error bars represent model parameter estimates and their 95 % bias-corrected confidence intervals (CIs). The number of study cases (*k*) is indicated below the error bars. Black dots correspond to natural herbivory, and white dots to simulated herbivory. The vertical dashed line at zero represents the null hypothesis (no difference between control and herbivore-damaged plants). Effects are considered significant when the 95 % CI does not include zero.

Plant damage significantly reduced floral traits, pollinator attraction, and plant reproductive success (*Q*_M_ = 15.41, *P* < 0.001; Fig. 1). Both the type of damage (*Q*_M_ = 10.31, *P* = 0.006) and the plant tissue affected (*Q*_M_ = 22.97, *P* = 0.003) exerted significant effects on these responses (Fig. 2). Notably, the magnitude of the impact was strongly moderated by the interaction between damage type and the tissue affected (*Q*_M_ = 27.91, *P* < 0.001). Specifically, floral traits were significantly reduced by natural (but not simulated) leaf damage and by simulated damage to flowers and stems (Fig. 2). Pollinator attraction declined in response to both natural and simulated leaf damage, as well as due to natural flower damage, but remained unaffected by simulated flower damage (Fig. 2). Plant reproduction was significantly reduced by natural damage to both leaves and flowers (Fig. 2). Lastly, root damage and mixed damage (regardless of type) showed no significant effects on floral traits, pollinator attraction or reproductive success (Fig. 2). Full statistical results are provided in Supplementary Data Table S1 of Appendix S2.

## DISCUSSION

Herbivory and pollination are key ecological processes that jointly shape plant reproductive outcomes (Underwood *et al*., 2020). Our expanded meta-analysis, which included more than twice the number of study cases compared to Moreira *et al*. (2019), confirmed their finding that herbivore damage generally reduces flower production/quality, diminishes floral attractiveness to pollinators, and decreases plant reproductive success. Nonetheless, these negative effects were dependent on the specific plant tissue damaged and the type of herbivory, mirroring several patterns observed in the original study. Specifically, we recovered the following trends reported by Moreira *et al*. (2019): natural leaf damage significantly reduced floral traits, pollinator attraction, and plant reproduction; natural flower damage decreased pollinator attraction; and root damage had no significant overall effect. On the other hand, our results also reveal important differences. Most notably, we found that natural flower damage significantly reduced plant reproductive success, a pattern not detected in the earlier study (Table 1). In addition, we newly observed that simulated flower damage reduced floral traits and that simulated leaf damage negatively affected pollinator attraction, though neither one affected plant reproduction (Table 1). In contrast, Moreira *et al*. (2019) reported no significant effects of simulated herbivory on any response variable (Table 1). Our analysis also incorporated two new categories not evaluated in the previous meta-analysis: stem damage and mixed tissue damage. While mixed damage (regardless of herbivory type) had no significant effect on any response variable, simulated – but not natural – stem damage significantly reduced floral traits, but without affecting pollinator visitation or plant reproductive success.

The specific plant tissue affected by herbivory is a critical factor to consider in studying herbivore–pollinator interactions and plant fitness outcomes, as it could modulate the occurrence and strength of direct or plant-mediated interactions (e.g. direct interference effects via physical proximity of herbivores and pollinators, or endogenous plant processes among vegetative and reproductive tissues). Our expanded dataset corroborates the conclusion by Moreira *et al*. (2019) that root herbivory has no significant net effect on floral traits, pollinator attraction or plant reproductive success. Despite increasing the number of study cases addressing root damage from 25 to 38, the overall pattern remained unchanged. This result aligns with previous literature documenting highly variable and often species-specific outcomes of root herbivory (Poveda *et al*., 2003; Knochel *et al*., 2010; Barber *et al*., 2015; Ghyselen *et al*., 2016; Rusman *et al*., 2018). Thus, the continued lack of a generalizable effect suggests that the influence of root herbivory on pollination (expectedly indirect) and plant reproduction (could involve both direct and indirect effects) may be limited in scope or be highly context-dependent. By contrast, natural damage to leaves and flowers consistently produced negative effects (potentially acting via direct and/or indirect pathways), significantly reducing floral attractiveness, pollinator visitation, and plant reproductive success – confirming patterns originally reported in Moreira *et al*. (2019). The increased sample size in our analysis strengthened the certainty of these patterns. Furthermore, we detected a significant reduction in plant reproductive output associated with natural damage to flowers, an effect that was previously non-significant and slightly positive in Moreira *et al*. (2019)(see Table 1), which could involve both direct (e.g. herbivore–pollinator interference, reduced allocation to fruit-filling) or indirect (via reduced pollinator attraction, though the lack of effect on floral traits by floral herbivory suggests this type of effect may be weak) pathways. This shift highlights the sensitivity of this particular test to sample size, probably due to the low number of study cases (*n* = 17) in the earlier meta-analysis. Together, our findings highlight the importance of adequately representing each combination of type of tissue damaged and response category to accurately assess herbivore impacts on plant reproduction within the context of pollination success.

A key novel finding of this meta-analysis is that simulated herbivory produced negative effects on floral traits and pollinator attraction comparable to those of natural herbivory. By contrast, the previous synthesis by Moreira *et al*. (2019) reported no significant effects of simulated damage to these tissues, with effect sizes that were a mix of positive and negative but statistically non-significant. Specifically, our updated analysis reveals that simulated leaf damage significantly reduces pollinator attraction, while simulated flower and stem damage reduce floral traits. These newly detected effects are probably attributable to increased sample sizes in the updated meta-analysis, which enhanced statistical power. For instance, the number of study cases assessing the effect of simulated leaf damage on pollinator attraction increased by 200 %, and the number of cases assessing simulated flower damage on floral traits increased by 21 % (Table 1). These results underscore the value of updating meta-analyses to uncover patterns that may remain undetected in earlier, underpowered datasets.

Herbivory in natural systems is highly diverse and can affect all plant organs. Notably, many economically important agricultural pests are stem-boring species (e.g. Taveras *et al*., 2004; Perish *et al*., 2023), and removal of apical meristems is among the most well-documented cases of herbivory-induced overcompensation (Garcia and Eubanks, 2019). Our synthesis revealed that simulated – but not natural – stem damage significantly reduced floral traits. Although natural stem damage did not influence pollinators and no studies with simulated stem damage assessed the effects on pollinator attraction, this result suggests that stem herbivory may strongly influence flower production and could indirectly affect pollinator visitation. Stem damage can limit flowering by reducing resource allocation to reproductive processes (Dube *et al*., 2019; Conrad *et al*., 2021). Conversely, it may trigger overcompensatory responses, such as increased flowering (Lennartsson *et al*., 2018; Garcia and Eubanks, 2019), potentially enhancing pollinator visitation (Lay *et al*., 2011). That said, given that natural stem damage showed no significant effects, these findings indicate that the consequences of stem herbivory are probably context-dependent and not consistently generalizable (e.g. contingent on methodological effects) – similar to patterns observed for root herbivory.

Mixed damage, defined here as damage to multiple plant tissues simultaneously (e.g. grazing that affects both leaves and flowers), also failed to significantly affect any response variable. This is notable given that some of the individual tissue types included in this category (e.g. leaves and flowers) showed significant negative effects on floral traits and pollinator attraction when damaged in isolation. While the mechanisms underlying the effects of mixed damage remain difficult to disentangle, they are ecologically relevant, as many generalist herbivores feed across multiple plant tissues (Ali and Agrawal, 2012). Understanding how mixed damage influences plant–pollinator interactions and fitness is particularly pertinent in natural and agricultural systems (Freeman *et al*., 2003; Benning and Moeller, 2019). Despite their ecological importance, the lack of significant effects here suggests that generalist herbivory, when pooled into a single mixed-damage category, may not consistently mediate plant–pollinator outcomes. Grazing, one form of mixed damage, is well known to shape both plant and pollinator communities (Allan, 2022). Yet, the broad-scale nature of such interactions may obscure specific, tissue-level effects on reproductive traits in meta-analytical frameworks. As with stem damage, further research is needed to assess how sensitive these conclusions are to sample size and to clarify the ecological and evolutionary consequences of damage impacting multiple plant tissues.

Overall, our updated meta-analysis reinforces the critical role herbivory has in shaping floral traits, pollinator attraction, and plant reproductive success, while also highlighting how these effects depend strongly on the type of tissue damaged and whether damage is natural or simulated. Future research should aim to disentangle the mechanisms underlying tissue-specific herbivory effects, particularly for stem and mixed damage, where our results suggest context-dependent impacts that remain poorly understood. Importantly, our findings also demonstrate the validity of using artificial damage to realistically assess these dynamics under experimental contexts where natural herbivory is difficult to manipulate. Future studies are needed that conduct damage to two or more tissues (e.g. floral and leaf factorially), measure both plant vegetative and reproductive traits, and record pollinator numerical and behavioural responses to add a more nuanced understanding of the mechanisms behind herbivore–plant–pollinator interactions (e.g. direct interference by herbivores or plant-mediated indirect effects on pollinators) and reductions in plant reproductive success (e.g. direct tissue loss or indirectly via changes in pollinator attraction or efficiency). This could also include manipulations of plant resource availability (e.g. via fertilization) and pollination manipulations to explore in more detail the roles of resource allocation and pollen limitation. More broadly, longer-term studies tracking the temporal dynamics of herbivory and pollination across plant ontogeny would improve our understanding of compensatory responses and their influence on reproductive outcomes. Finally, there is a need to expand research across diverse plant functional types and ecosystems, including underrepresented agricultural systems such as tropical fruit orchards, smallholder cereal fields, and legume-based intercropping systems, as well as natural systems like arid shrublands, alpine grasslands, and mangrove forests. Broadening the scope to these contexts will increase inference and help explore mechanistic variability as a function of different biotic (herbivore or plant types) and abiotic (e.g. nutrient or water limitation) conditions. Combined, these approaches will allow future studies to yield predictive insight into how herbivory shapes plant reproductive ecology and the resilience of plant–pollinator networks in changing environments.

## Supplementary Material

mcaf258_Supplementary_Data
