# Supplementary material for: The effect of herbivory on pollinators: a revisited meta-analysis
Source: Ann Bot. 2025 Oct 16;137(4):879–85. doi: 10.1093/aob/mcaf258 (PMC13095882; doi:10.1093/aob/mcaf258)
Supplement: mcaf258_Supplementary_Data [file mcaf258_supplementary_data.zip › Appendix S1.docx]

## Appendix S1. Sensitivity and bias analysis

The 171 primary studies contributed between 1 and 68 study cases, raising the possibility that some studies could disproportionately influence the meta-analysis results. To assess the robustness and impartiality of our findings, we performed two complementary sensitivity analyses. First, we reran the main analysis—a multi-level random-effects meta-analysis testing differences among response variables and induced tissues—while sequentially excluding one primary study at a time. This approach evaluated whether effect size estimates were driven by particularly influential studies, especially those contributing many cases. Across the 171 iterations, each corresponding to the exclusion of one primary study, we compared model parameter estimates for each herbivory treatment (control vs. herbivore-damaged) to confirm their consistency regardless of individual study inclusion (Fig. S1).


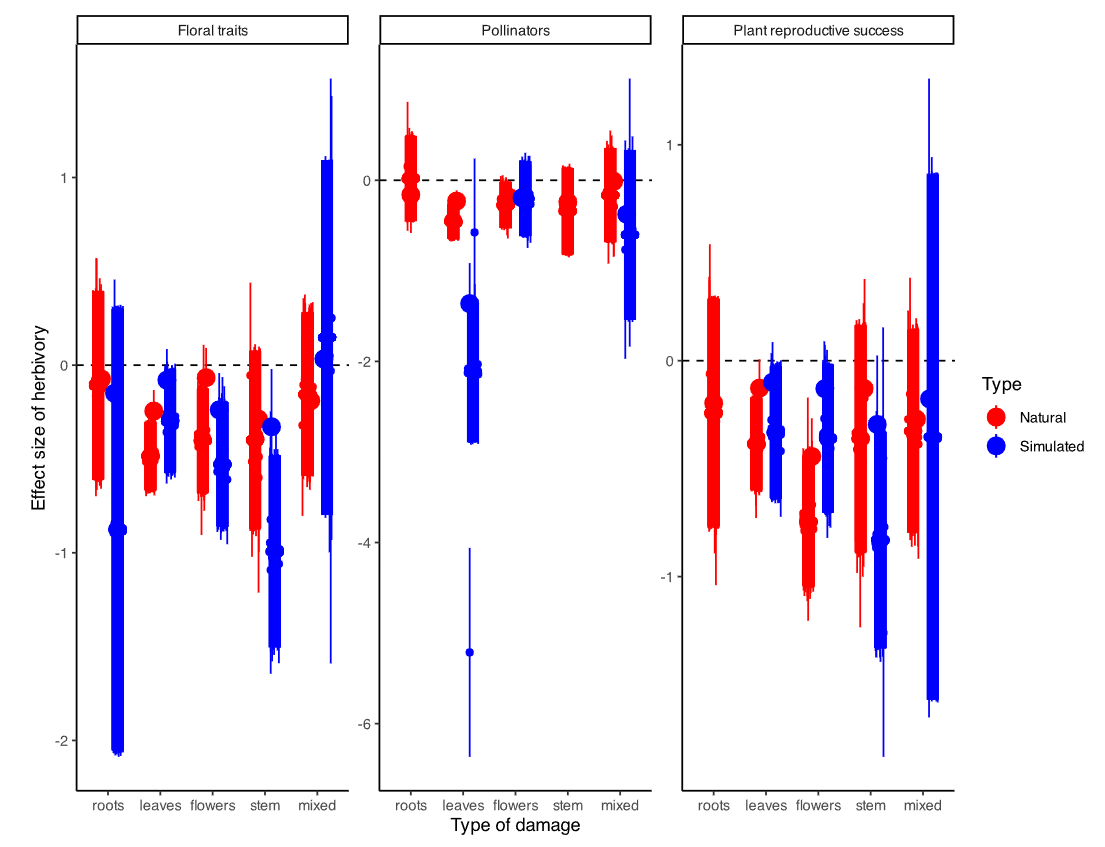


**Figure S1.** Sensitivity analysis in which each study was removed sequentially and the analysis rerun. Small dots with lines represent effect sizes and their 95% confidence intervals for each iteration, while large dots indicate the mean effect size from the main analysis.

Second, to account for potential bias from multiple study cases originating within the same primary study, we implemented a random sampling procedure in which one study case per study was selected at random and the main analysis was rerun as a random-effects meta-analysis. This process was repeated 1,000 times. For each iteration, we extracted the estimated effect size for each herbivory treatment and compared these estimates to those from the main analysis within the 95% distribution of the 1,000 samples (Fig. S2). Both sensitivity analyses converged on the same conclusion: resampling one study case per primary study produced a 95% distribution of effect sizes that closely overlapped with the mean effect size and confidence interval from the multi-level random-effects meta-analysis (Fig. S1, S2). Together, these results reinforce the robustness and reliability of the findings presented in this study.
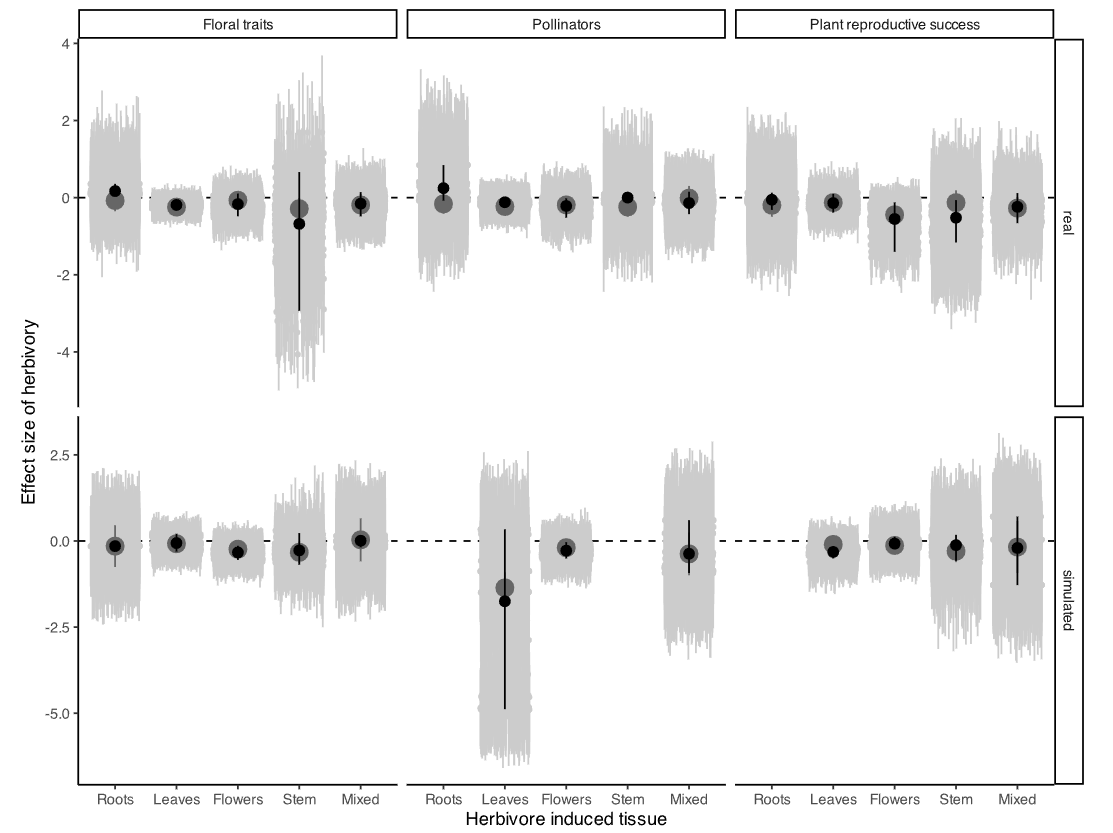


**Figure S2.** Sensitivity analysis in which one study case per study was randomly selected to perform the random-effects meta-analysis. This random sampling was repeated 1,000 times. Gray lines represent the 95% confidence intervals from these trials, while the black dot with error bars indicates the grand mean and 95% confidence interval across all trials. The large dark gray dot represents the mean from the main analysis.

The funnel plot appeared symmetrical, suggesting that studies with higher precision tended to report effect sizes close to zero, whereas larger effect sizes were more often associated with lower-precision studies (Fig. S3). The cumulative meta-analysis showed considerable variability in effect sizes during the earlier years, particularly in the 1990s, followed by a gradual decline in variability throughout the 2000s, with estimates stabilizing in the 2010s and 2020s (Fig. S4). Beyond the expected increase in precision over time, there was little evidence of a consistent temporal trend in effect sizes.

**
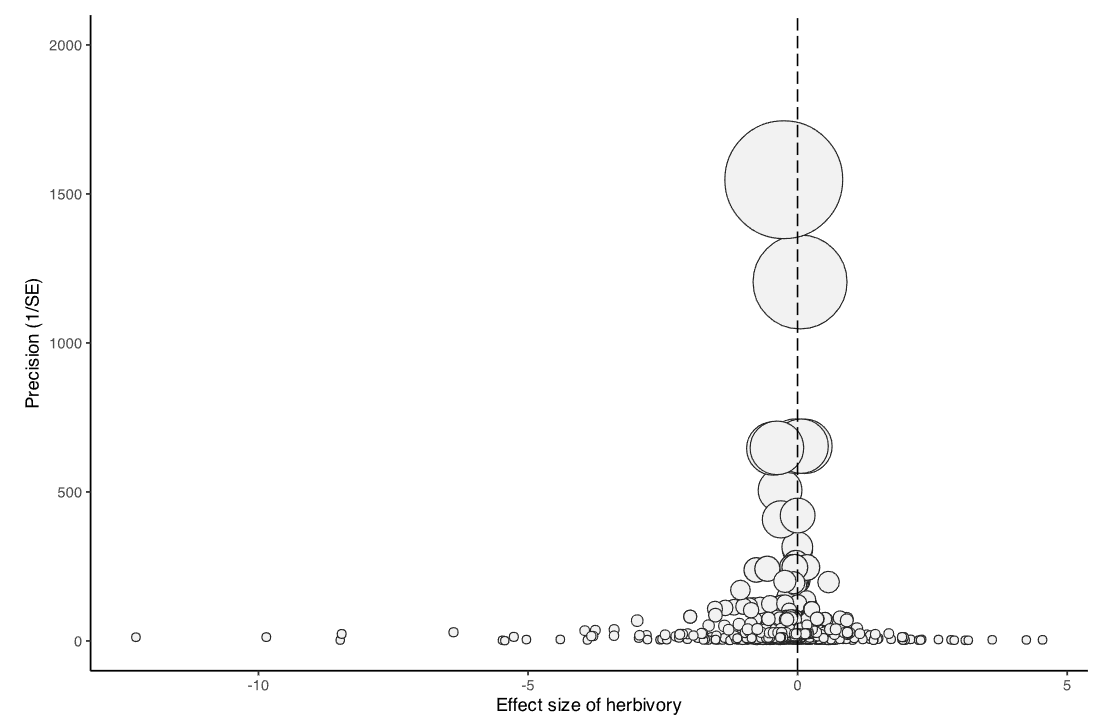
**

**Figure S3.** Funnel plot illustrating the relationship between effect size and precision, with dot size proportional to the inverse of the standard error.


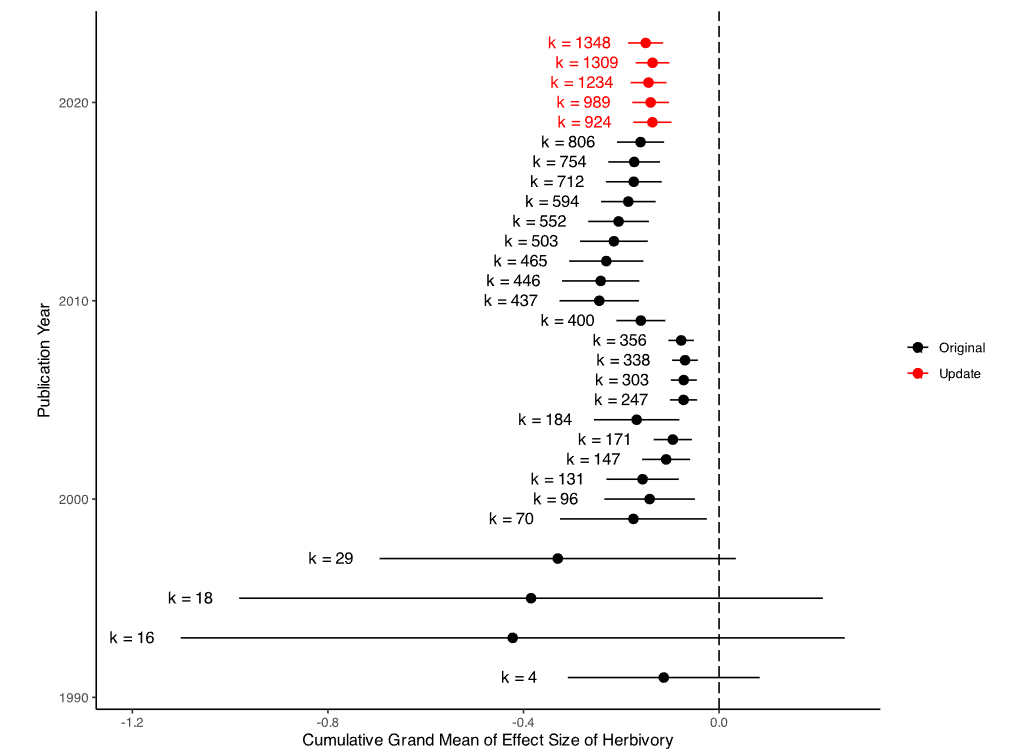


**Figure S4.** Cumulative mean effect size of herbivory on reproduction across years. The dashed vertical line indicates no effect of herbivory. Bars represent 95% confidence intervals, and k denotes the cumulative number of study cases. Publications prior to 2019 (from Moreira et al. 2019, Haas and Lortie 2020) are shown in black, whereas publications from the updated search are shown in red.

**REFERENCES**

Haas, S. M., and C. J. Lortie. 2020. A systematic review of the direct and indirect effects of herbivory on plant reproduction mediated by pollination. PeerJ **8**:e9049.

Moreira, X., B. Castagneyrol, L. Abdala-Roberts, and A. Traveset. 2019. A meta-analysis of herbivore effects on plant attractiveness to pollinators. Ecology **100**:e02707.
