# Supplementary material for: The effect of herbivory on pollinators: a revisited meta-analysis
Source: Ann Bot. 2025 Oct 16;137(4):879–85. doi: 10.1093/aob/mcaf258 (PMC13095882; doi:10.1093/aob/mcaf258)
Supplement: mcaf258_Supplementary_Data [file mcaf258_supplementary_data.zip › Appendix S2.docx]

**Table S1.** Statistics (Q_M_ and *p*-value) for type of measurement (floral traits, pollinator attraction, and plant reproductive success), tissue damaged (roots, leaves, flowers, stem, mixed), type of damage (natural or simulated), and their double and triple interactions. Significant *p*-values (*p* < 0.05) are shown in bold.

| Moderators | Q_M_ | *p*-value |
| --- | --- | --- |
| Measurement | 15.41 | **<0.001** |
| Tissue | 9.42 | 0.051 |
| Damage type | 6.33 | **0.012** |
| Measurement × tissue | 22.97 | **0.003** |
| Measurement × damage type | 10.31 | **0.006** |
| Tissue × type | 5.69 | 0.223 |
| Measurement × tissue × damage type | 27.91 | **<0.001** |
